# Comparing Relapse Rates in Real-World Patients With Schizophrenia Who Were Considered Adequately Versus Not Adequately Treated With Paliperidone Palmitate 1-Month or 3-Month Before Transitioning to the 6-Month Formulation

**DOI:** 10.1192/j.eurpsy.2026.10518

**Published:** 2026-07-31

**Authors:** J. Mehawej, M. Daskiran, J. Antunes, M. Wahba, C. Obando, L. Killion, K. Knight, I. Turkoz

**Affiliations:** 1 Johnson & Johnson, Titusville, NJ; 2 Johnson & Johnson, Raritan, NJ, United States; 3 Johnson & Johnson, Porto Salvo, Portugal; 4 Johnson & Johnson, Cairo, Egypt; 5 Johnson & Johnson, Horsham, PA, United States

## Abstract

**Introduction:**

Paliperidone palmitate (PP) 1-month (PP1M), 3-month (PP3M), and 6-month (PP6M) formulations are long acting injectables (LAIs) that have been proven to be effective in maintaining functional and symptomatic control, delay time to relapse, and reduce the risk of relapse in patients with schizophrenia. Following the label or product insert when transitioning patients between the three available PP extended-duration LAI formulations supports favorable patient outcomes.

**Objectives:**

To evaluate real-world data on relapse rates in adult patients with schizophrenia who were adequately treated (AT) versus not adequately treated (NAT) with PP1M or PP3M before transitioning to PP6M.

**Methods:**

Data from the Merative™ MarketScan^®^ Multi-State Medicaid Database were analyzed. The study included adults aged ≥18 years who had ≥1 claim for a schizophrenia diagnosis and maintained continuous medical and prescription coverage for at least three months before and/or after the index date of PP6M. Patients were classified into two groups: those considered AT, defined as having received ≥4 PP1M doses or ≥1 PP3M dose, with the last dose of PP1M or PP3M corresponding to index PP6M dose; and those NAT, defined as patients who received ≤3 PP1M dose or no PP3M doses. A patient was considered relapsed if any of the following claims record was present during the 12-month study period: mental health–related inpatient hospitalization, suicide attempt, suicidal ideation, homicidal ideation, intentional self-harm/poisoning, and violent behavior. Relapses were captured and the differences in risk between the groups were calculated along with corresponding 95% confidence intervals (CIs).

**Results:**

Among 586 patients treated with PP6M, 282 met the inclusion criteria, with 165 classified as AT and 117 as NAT. The majority of patients were male (69.5%), and the mean age was 38.9 years. The relapse rate was lower in the AT cohort (7.3%) compared to the NAT cohort (12.8%). Most relapses were due to mental health-related inpatient hospitalization. The risk difference in relapse rates was ˗5.56%, (95% CI, -12.8% to 1.7%).

**Conclusions:**

In this real-world Medicaid insurance claims cohort, relapse rates were low among patients on PP6M, with those being adequately treated with PP1M or PP3M prior to transitioning experiencing even lower relapse rates, highlighting the potential benefit of appropriate treatment before switching to the longer-acting formulation.

**Disclosure of Interest:**

J. Mehawej Shareholder of: Johnson & Johnson, Employee of: Johnson & Johnson, M. Daskiran Shareholder of: Johnson & Johnson, Employee of: Johnson & Johnson, J. Antunes Shareholder of: Johnson & Johnson, Employee of: Johnson & Johnson, M. Wahba Shareholder of: Johnson & Johnson, Employee of: Johnson & Johnson, C. Obando Shareholder of: Johnson & Johnson, Employee of: Johnson & Johnson, L. Killion Shareholder of: Johnson & Johnson, Employee of: Johnson & Johnson, K. Knight Shareholder of: Johnson & Johnson, Employee of: Johnson & Johnson, I. Turkoz Shareholder of: Johnson & Johnson, Employee of: Johnson & Johnson.

